# Developing Scenario‐Based Strategies for Health, Climate, and Environmental Preparedness: The One Health, One Earth Approach

**DOI:** 10.1029/2025GH001674

**Published:** 2026-04-01

**Authors:** Azar M. Abadi, Kyle D. Brumfield, Moiz Usmani, Gemma Jiang, T. C. Chakraborty, Yuzhou Wang, Zhijie Zhou, Cenlin He, Minghao Qiu, Jennifer D. Stowell, Douglas Fritz, Sunil Kumar, Qiang Guo, Ying Xiong, Georgia Destouni, Qian Xiao, Xindi C. Hu, Kelvin C. Fong, Yun Hang, John Nielsen‐Gammon, Rima Habre, Jennifer R. Bratburd, Christopher W. Tessum, Antarpreet Jutla, Rita R. Colwell, Gabriel M. Filippelli, Thanh H. Nguyen

**Affiliations:** ^1^ Environmental Health Sciences Department University of Alabama at Birmingham Birmingham AL USA; ^2^ Department of Cellular Biology and Molecular Genetics University of Maryland College Park MD USA; ^3^ University of Maryland Institute for Advanced Computer Studies University of Maryland College Park MD USA; ^4^ Department of Civil, Construction and Environmental Engineering University of Alabama at Birmingham Birmingham AL USA; ^5^ Institute for Research in the Social Sciences Colorado State University Fort Collins CO USA; ^6^ Pacific Northwest National Laboratory Richland WA USA; ^7^ Zachry Department of Civil and Environmental Engineering Texas A&M University College Station TX USA; ^8^ Department of Civil and Environmental Engineering University of California Berkeley CA USA; ^9^ Department of Geography and Geographic Information Science University of Illinois Urbana‐Champaign Urbana IL USA; ^10^ Research Applications Laboratory NSF National Center for Atmospheric Research Boulder CO USA; ^11^ School of Marine and Atmospheric Sciences Stony Brook University Stony Brook NY USA; ^12^ Program in Public Health Stony Brook University Stony Brook NY USA; ^13^ Global Environmental, and Occupational Health University of Maryland School of Public Health College Park MD USA; ^14^ Department of Genetics University of Cambridge Cambridge UK; ^15^ Department of Environmental Engineering Sciences Herbert Wertheim College of Engineering University of Florida Gainesville FL USA; ^16^ Institute of Systems and Information Engineering University of Tsukuba Tsukuba Japan; ^17^ Tsukuba Institute for Advanced Research (TIAR) University of Tsukuba Tsukuba Japan; ^18^ Guangdong‐HongKong‐Macau Joint Laboratory of Collaborative Innovation for Environmental Quality College of Environment and Climate Institute for Environmental and Climate Research Jinan University Guangzhou China; ^19^ Department of Climate and Space Sciences and Engineering University of Michigan Ann Arbor MI USA; ^20^ Department of Physical Geography Stockholm University Stockholm Sweden; ^21^ Department of Sustainable Development Environmental Science and Engineering KTH Royal Institute of Technology Stockholm Sweden; ^22^ Department of Epidemiology School of Public Health University of Texas Health Science Center at Houston Houston TX USA; ^23^ Department of Environmental and Occupational Health George Washington University Washington DC USA; ^24^ Department of Environmental and Occupational Health Sciences School of Public Health University of Texas Health Science Center at Houston Houston TX USA; ^25^ Department of Atmospheric Sciences Texas A&M University College Station TX USA; ^26^ Keck School of Medicine and Spatial Sciences Institute University of Southern California Los Angeles CA USA; ^27^ University of Wisconsin‐Madison Madison WI USA; ^28^ Department of Civil and Environmental Engineering University of Illinois at Urbana‐Champaign Urbana MI USA; ^29^ Environmental Resilience Institute Indiana University Bloomington IN USA; ^30^ Department of Civil and Environmental Engineering University of Illinois at Urbana‐Champaign Urbana IL USA

**Keywords:** human centered design, community, climate extremes, One Health, environmental health

## Abstract

Climate change amplifies many threats to human health. Despite advances in understanding climate change dynamics and impacts, there remains a critical gap in translating scientific knowledge into equitable, and community‐driven health interventions. The inaugural *One Earth*, *One Health* workshop sought to explore this gap through human‐centered design exercises involving interdisciplinary researchers from climate and Earth sciences, engineering, epidemiology, microbiology, and environmental health. Although participants did not co‐develop solutions with affected communities, they used stakeholder role‐playing to guide ideation and lay groundwork for actionable plans. Through these methods, participants identified community needs and proposed prototype solutions to alleviate health threats exacerbated by global environmental change. Prototypes were organized around infectious diseases, extreme weather, and air quality, as illustrative themes rather than an exhaustive set of risks. Key solutions included strategies for anticipatory systems and early warning (e.g., integrating environmental signals with health data), inclusive communication and infrastructure needs for responding to extreme weather events, and integrated platforms visualizing air quality trends to support tailored, context‐aware guidance beyond one‐size‐fits‐all alerts. The workshop highlighted opportunities such as leveraging machine learning, Earth observation, and real‐time surveillance to protect communities, but also noted barriers including data quality, technological redundancy, privacy, and governance challenges. Additionally, participants emphasized the need for interdisciplinary teams capable of collaborating across sectors, breaking down silos and addressing gaps in training and education. Overall, the workshop illustrates how process‐driven, human‐centered approaches can help surface user needs and generate testable prototype concepts, while underscoring the importance of direct community partnership for implementation.

## Plain Language Summary

Climate and other environmental changes amplify threats to human health, such as extreme weather events, infectious diseases, and poor air quality. When trying to understand which hazards and exposures pose the risk to human health, which populations are most vulnerable, and what interventions might be most protective, scientists rely on hypothesis‐driven approaches. Such approaches may not directly reflect the lived experiences or priorities of affected communities. The *One Earth*, *One Health* workshop congregated researchers across disciplines to test a method called human‐centered design. Although this workshop did not include direct participation from every key stakeholder groups, participants role‐played as community members, such as healthcare workers, city planners, parents, and concerned citizens, to simulate more inclusive solution development. Participants discussed and co‐developed early‐stage, user‐centered solutions, such as better disease prediction tools, clearer emergency communications, and unified platforms for air quality monitoring and alerts. Although promising, the solutions face multiple challenges, including limitations in data availability, timeliness, and interoperability and technological complexity. The workshop underscored the importance of collaboration and co‐creation as guiding principles for future climate‐health research and intervention design, including the need to engage researchers, policymakers, healthcare workers, and communities in subsequent phases to support practical, equitable, and beneficial outcomes.

## Key Points

- Human‐centered design helped translate stakeholder perspectives into practical, cross‐sector solution concepts related to health risks
- Human‐centered workshops generated actionable, community‐informed health resilience prototypes grounded in data and early warning
- Participants emphasized that implementation depends on reliable data, governance, funding, policy support, and community partnerships

## 1 Introduction: The Design Challenge

Climate and other global changes amplify human health risks across multiple fronts, including slowing improvement of air and water quality, increasing the rates of emergence and spread of infectious diseases, and increasing the frequency and severity of extreme weather events (Mora et al., 2022; Romanello et al., 2022). A prime example is worsening air quality indirectly, driven by more frequent wildfires that alters atmospheric conditions and increases energy demand and emissions through greater cooling needs (Bolan et al., 2024; Filonchyk et al., 2022). Addressing these interconnected challenges requires both scientific innovation and a shift in how research is conceptualized, designed, and translated into action. Traditional research approaches, while valuable, often prioritize basic or hypothesis‐driven inquiry that advances physical understanding but may not directly address the immediate concerns or lived experiences of affected communities (Lorsch, 2014). There is growing recognition that integrating community‐centered methods, such as participatory design or co‐creation, can complement traditional science by enhancing relevance, equity, and implementation of interventions (Agnello et al., 2025; Wallerstein & Duran, 2010).

Effectively translating climate–health science into action also requires engagement beyond the natural and health sciences. Disciplines such as the social sciences, humanities, arts, and music contribute essential insights into communication, trust‐building, cultural context, narrative framing, and behavior change—factors that strongly influence whether interventions are understood, accepted, and sustained. However, integrating these approaches into climate and health research requires deliberate testing of methods, shared language, and feasibility within interdisciplinary scientific teams before they can be responsibly applied in community settings.

In response to these gaps and opportunities, the first annual *One Earth, One Health* workshop in 2024 convened early‐career and senior researchers across geosciences, climate science, epidemiology, microbiology, and environmental health to examine challenges in designing human‐centered solutions. This initial workshop was intentionally structured as a pilot, process‐driven convening among researchers, with the goal of evaluating whether and how human‐centered design principles could be operationalized within climate–health research workflows. The *One Earth, One Health* framework is distinctive in its ambition to integrate Earth system science and public health into a unified, actionable model (Danasekaran, 2024). This approach helps reduce institutional fragmentation by aligning environmental, health, and community priorities early in the design process.

Rather than being hypothesis‐driven, the workshop was intentionally process‐driven, with participants exploring the perspectives and potential needs of community stakeholders, such as parents, healthcare workers, city planners, and the general public, who are affected differently by climate‐related health threats. Although participants were exclusively researchers, they role‐played user personas to support structured perspective‐taking during an early design phase, recognizing that such approaches cannot substitute for lived experience and require validation through direct stakeholder engagement. This “storyline” approach can be helpful for developing more realistic scenarios of environmental exposures and impacted groups (Chakraborty et al., 2025). Through structured activities modeled after established design sprints, a time‐constrained, iterative method used to rapidly generate and prototype solutions (Knapp et al., 2016). Briefly, teams developed user stories (Step 1: Empathize), generated solution ideas (Step 2: Ideate), mapped opportunities by importance and feasibility to create prototype concepts for user‐centered interventions (Step 3: Prototype) and identified future directions (Step 4: Reflection).

The process emphasized designing “with” stakeholders rather than “for” them, ensuring that proposed solutions reflect personal experiences, local context, and practical constraints. This approach reinforced principles of co‐creation, trust‐building, accessibility, and equity as guiding values, even though direct engagement with community partners did not occur during this phase. Recognizing the importance of communication, narrative, and cultural context, subsequent workshops are explicitly designed to incorporate expertise from communication scholars and related disciplines, alongside direct engagement with community members, stakeholders, and decision‐makers. By the workshop's end, participants had collaboratively built a portfolio of suggested prototype solutions aimed at making human societies more resilient to global change. These efforts were organized around three themes: (a) infectious diseases, (b) extreme weather, and (c) air quality.

## 2 Workshop Design

**Figure 1:**
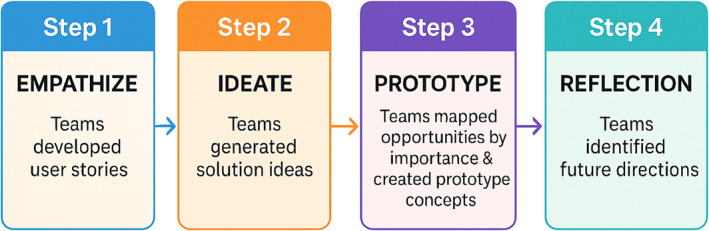
Design thinking process: A human‐centered approach guiding teams through empathy, ideation, prototyping, and reflection to develop impactful solutions.

A total of 117 participants attended the workshop and were assigned to one of eight breakout groups organized around three themes of infectious diseases (three groups), air quality (three groups), and extreme weather (two groups). Within each group, participants identified key user needs, design challenges, and strategic opportunities to advance actionable solutions at the intersection of climate, health, and community well‐being. The resulting design challenges highlighted a shared need for anticipatory systems, inclusive communication strategies, and user‐centered tools to help narrow the disconnect between global change science and health action. While the strategies were hypothetical, they offer insight into how cross‐disciplinary dialog, framed through a human‐centered design lens, can inform future solution development. The process is summarized in Figure 1.

### 2.1 Empathize

In this storytelling step, participants were invited to focus on a single character or role, explore goals and motivations to develop a user story employing the following:

“**As a** (e.g., city planner), **I want to** (e.g., design green spaces, cooling zones, and air‐quality monitoring systems in urban areas), **so that I can** (e.g., reduce heat‐related illnesses and protect vulnerable populations from heat and pollution spikes during heatwave extremes).” Each participant was first invited to individually brainstorm as many user stories as possible, before the group converged on a single story per table for the following:

#### 2.1.1 Infectious Diseases

Breakout discussions described a future where health systems proactively manage infectious disease threats using methods and tools that integrate environmental signals with health data and community needs. For example, participants discussed how hypothetical users, such as bus drivers and tour guides, might benefit from clear guidance to protect themselves while contributing to local economies. Medical students and healthcare providers may rely on up‐to‐date training and exposure forecasts to deliver effective care. Policymakers and insurers may use localized data to identify vulnerable populations and allocate resources. City planners could use disease risk maps to guide infrastructure decisions, while educators and communicators may help translate complex science for public understanding. A human‐centered approach may offer a structured process for identifying and addressing diverse user needs, though further evaluation with stakeholders is necessary to validate these proposed benefits.

#### 2.1.2 Extreme Weather

Participants envisioned a future where communities can rapidly and equitably respond to extreme weather events, requiring systems that merge weather and climate forecasting, inclusive communication, and access to life‐saving infrastructure. Public health officials and first responders need real‐time dashboards integrating environmental and health data, while diverse groups, including residents with limited resources or local knowledge (e.g., low‐income households, visitors, tourists, non‐native speakers), may benefit from multilingual alerts and transportation support during emergencies. Healthcare providers benefit from health records temporally and spatially linked to climate and weather exposures (e.g., heat, air quality, extreme events) to anticipate and address heat‐related illnesses. City planners can use predictive models to guide investments aimed at reducing exposure risks. In this context, a human‐centered framework could be used to explore how extreme weather risks are expected differently across communities and to identify intervention points that support earlier and more equitable health protection, though additional empirical evidence would be needed to substantiate this approach.

#### 2.1.3 Air Quality

The groups articulated a vision in which improving air quality resilience requires tools that integrate diverse data sources without overwhelming users or duplicating existing platforms. People from all walks of life including residents, parents, scientists, healthcare providers, and policymakers are increasingly concerned about the impacts of air quality and specific environmental exposures, such as wildfire smoke, allergens, and industrial emissions. From factory owners aiming to reduce emissions cost‐effectively to school administrators seeking best practices to manage indoor air pollution, the demand for real‐time, accessible environmental data was noted in many applied public health efforts (Kearney et al., 2015). Participants proposed hypothetical user needs ranging from mobile alerts for caretakers and healthcare providers, to exposure forecasts for workers and transportation planners.

The discussions emphasized a shared desire for intuitive, real‐time data visualization tools that synthesize satellite, sensor, and model data, validated for reliability and usability. Some groups proposed platforms that leverage emerging analytical approaches (e.g., predictive ML models and exploratory use of large language models (LLMs)) for personalized guidance, such as localized health tips, tailored outputs for concerned groups rather one alert for all, incorporating local knowledge, behavioral constraints, and language. Concerns included sustainable funding, data privacy, public trust, and technical complexity. For example, LLMs may generate inaccurate or non‐contextual advice in critical health scenarios (Oviedo‐Trespalacios et al., 2023). Additionally, ML systems may have higher energy demands and environmental impacts than more targeted solutions (Eilam et al., 2024; Henderson et al., 2020). Thus, a balanced approach that is accurate, equitable, and easy to maintain is needed to address worsening air pollution and related health concerns. Similar considerations apply to other forms of environmental pollution, including water and soil contaminants.

### 2.2 Ideate

Participants began by rapidly generating a wide range of solution ideas individually, a process designed to encourage uninhibited creativity at the outset of ideation, each captured on a sticky note. They then shared these ideas in their design teams, making quick connections and asking exploratory questions to surface potential synergies. As a group, they mapped emerging strategic opportunities on an Importance/Difficulty Matrix, assessing each idea's value to users and feasibility of implementation. From this, they selected a “BIG EASY,” a high‐impact, low‐effort opportunity, as their design focus. Finally, they shared their strategic opportunity with two other teams for feedback and cross‐pollination. This process reflects a standard design thinking framework (Gonen, 2019), structured to move participants from divergent thinking to convergence, allowing teams to quickly generate, prioritize, and test user‐centered ideas.

#### 2.2.1 Infectious Diseases

Participants emphasized the potential of integrating machine learning with environmental surveillance to strengthen outbreak prediction, especially for zoonotic diseases sensitive to climate variability. ML‐driven models that draw on diverse data sets, such as phenology (seasonal crop, dust, and vegetation greening cycles), satellite observation, and public health records, can provide localized disease forecasts and early warning by identifying environmental conditions that influence disease transmission, such as vector breeding habitats, climate variability, and human exposure patterns (Farooq et al., 2022). Real‐time surveillance (e.g., vector traps, rapid sequencing, and wearable biometric devices), when integrated with demographic data, may also support more timely and precisely targeted warning and alert systems and broader public health responses (Ming et al., 2020). These strengths reflect a move toward anticipatory systems (Table 1). However, key limitations were also noted. Data quality control remains a persistent challenge, particularly when relying on community‐driven collection methods that may introduce bias, and when environmental data require extensive curation before they are suitable for public use. Limited accessibility and lack of interoperability across wearable biometric devices can hinder broader adoption and integration into public health systems (Seshadri et al., 2020). Moreover, overuse of alert systems can lead to public fatigue, diminishing the effectiveness of early warning (Baseman et al., 2013). Bias in model outputs and uneven regional coverage further complicate efforts to build equitable and effective predictive systems. Privacy and data governance concerns, particularly related to the use of personal identifiers in health and wearable data, were identified as key limitations for scalable and ethical implementation.

**Table 1:** Strengths and Gaps in Current Approaches to Global Change‐Driven Health Challenges

Topic of interest	Potential strategies	Expected limitations or barriers
Infectious disease	Ml‐driven outbreak predictions using satellite, phenology, and health data	Community‐driven data collection may introduce bias and inconsistency
	Real‐time surveillance integrated with sociodemographic risk stratification	Wearable tech adoption limited by cost and lack of integration with health systems
	Machine learning models enabling localized forecasts	Public fatigue from frequent alerts; limited trust in data‐driven systems
Extreme Weather	Using malls/libraries as informal cooling centers	Permission, liability, and operational concerns limit feasibility
	ML‐enhanced weather prediction integrated with health and infrastructure data	Generic alerts that fail to account for local risks and community needs
	Multimodal communication for reaching people with low digital access	Overdependence on smartphone‐based alerts excludes digitally marginalized communities
	Subsidized rideshare or mobile services for shelter access during extreme events	Inadequate transportation infrastructure or funding to operationalize access.
Air Quality	ML inclusive of LLMs reduce barriers for app development and enhance forecasting	Data validation between satellite and sensor data is labor‐intensive and costly. ML, especially LLMs, may have biased or for LLMs, hallucinated results
	Combining AQ data sets into one platform supports localized health messaging	New tools risk redundancy with existing applications (e.g., AirNow) and suffer low engagement
	Translating air pollution data into actionable health guidance	Interfaces may not be tailored to the need of diverse users; it is often unclear what cues or incentives will successfully prompt behavior change. Individual health guidance may be limited when collective or public policy approaches are needed for behavior change to reduce health risk

*Note.* The table contrasts promising strategies with known/expected limitations or barriers across these domains.

#### 2.2.2 Extreme Weather

Participants recommended leveraging existing infrastructure, such as malls and libraries, as cooling centers because of their familiarity and potential accessibility. Strategies like multimodal communication (sirens, radio, text) and subsidized rideshare programs were seen as valuable ways to reach underserved populations and bridge the transportation gaps. Machine learning‐enhanced forecasting with high spatial resolution was seen as a promising solution for issuing timely, accurate alerts at local/community scale. Nonetheless, these strategies come with limitations. Using private businesses as shelters poses logistical hurdles, including the need for formal agreements and concerns about liability and operating hours. Over‐reliance on digital alerts risks excluding those without smartphones or reliable internet access, and alerts often lack local context or specificity (Savoia et al., 2013). Participants emphasized the critical need for physical sirens and other non‐digital warning systems to reach people without phones, unhoused populations, and individuals in remote locations without cell service, underscoring the importance of maintaining an array of diverse early warning approaches. Transportation assistance is critical, but in many communities, infrastructure or funding may be inadequate.

#### 2.2.3 Air Quality

Participants saw strong value in integrating satellite, modeling, and ground monitoring data into a single user‐friendly platform to indicate the air quality trends and real‐time conditions. Some groups discussed layering air quality data into tools already used by the public (e.g., navigation applications, transit maps), although many of these features are already available in state‐of‐the‐art platforms (e.g., U.S. EPA, 2025). However, several challenges were noted. Many new tools risk duplicating existing functionality, leading to user fatigue and unclear differentiation (Karavas et al., 2021). Data validation, especially when discrepancies exist between remote sensing and ground sensors, remains a technical bottleneck. Tools are often not tailored to specific populations, quickly evolving situations, or contexts, limiting its potential for driving behavioral change. Privacy concerns and the need for iterative user testing also emerged as key issues.

### 2.3 Prototype

To prepare for user testing, each design team collaboratively created a storyboard illustrating their proposed solutions in response to given strategic design opportunity. Teams identified four to five key moments in the user journey to illustrate how a proposed solution might unfold in real‐world contexts. Teams also examined key assumptions embedded in their designs, asking critical questions to assess feasibility and inclusivity, such as whether cooling zones would be accessible and effective for all. The session concluded with teams rehearsing how to narrate their storyboard in the upcoming design exhibition.

A range of practical and innovative ideas emerged from this process. Although the questions can be broadly categorized into five sections (Table 2), most proposed solutions combination from two or more categories. Some solutions were low‐cost and highly actionable, such as designating existing malls and libraries as cooling centers, launching public awareness campaigns on heat safety, or adding risk alerts into commonly used navigation and weather applications. Other emerging solutions included machine learning models to forecast health impacts using climate, environmental, and socio‐demographic data, as well as real‐time, community‐based data collection to improve localized predictions.

**Table 2:** Key Interdisciplinary Research Questions That Emerged From the Collaborative Design Session, Organized Across Five Thematic Areas: ML and Data, Health Policy, Climate Impact, Behavior and Social, and Technology and Engagement

Sections	Topic of interest	Relevant research questions
ML and Data	Infectious disease	How can ML models improve forecasting accuracy while accounting for regional variations?
	Infectious disease	Can real‐time satellite and environmental data enhance the prediction of pathogen hotspots linked to land use changes?
	Extreme weather	How can real‐time satellite and sensor data improve neighborhood‐level risk prediction?
	Air quality	How to improve real‐time air quality prediction leveraging new satellite data (e.g., TEMPO, GEMS)? In what ways can ML models more effectively bridge the gaps between satellite data and ground‐level sensor inputs? Is ML an improvement over other data fusion techniques when factoring potential bias, data set limitations?
Health Policy	Infectious disease	What strategies are most effective for communicating health risks to diverse demographic groups?
	Infectious disease	What policies are needed to ensure ethical disease surveillance at the intersection of public health, agriculture, and conservation?
	Extreme weather	What are the most effective strategies for reaching people with limited access to digital communication?
	Extreme weather	What are the policy levers for integrating health, climate, and infrastructure planning?
Climate Impact	Infectious disease	How does climate‐driven crop phenology impact the timing of migratory bird stopovers and zoonotic disease transmission?
	Extreme weather	How do urban heat mitigation strategies affect health outcomes across socio‐demographic groups?
	Air quality	How does air pollution interact with climate/weather extremes? What are compounding health effects and effective mitigation methods of co‐exposure to climate extremes and air pollution?
Behavior and Social	Air quality	What effects do personalized air quality recommendations have on long‐term behavioral changes, such as choices in transportation and exercise routes? How can challenges to access data from a political standpoint be addressed?
	Air quality	Identify key pollutants that have a greater influence on population‐scale health and stress these pollutants more to the public or vulnerable groups
	Air quality	What are the major hurdles to reducing air pollution, especially pollution that affects large, at‐risk populations? How to effectively address air pollution concentration in those “high pollution days”?
Technology and Engagement	Air quality	Could incorporating large language models enhance user engagement while preserving transparency and trust, or will LLMs increase potential misinformation?
	Extreme weather	How can we scale up mobile cooling solutions in a cost‐effective way?

*Note.* Many of the proposed solutions, ranging from mobile‐based alerts to predictive machine learning models, cut across these domains and reflect integrated, real‐world applications.

A human‐centered approach to environmentally driven health management combines technological integration with stakeholder engagement through co‐creation (Tiller et al., 2021). Technological components can include machine learning models that forecast health impacts using climate, environmental, population movement, and sociodemographic data. Real‐time, community‐based data collection can support crowdsourcing of health symptoms and environmental conditions, improving localized predictive models (Alotaibi & Nassif, 2024; Colwell, 1996; Olawade et al., 2024). Machine learning models, paired with affordable diagnostic tools and sensors, may facilitate earlier warnings, including in resource‐limited settings (Poushter et al., 2022; Topol, 2019). Wearable biometric devices allow for personalized health monitoring, alerting individuals to environmental exposure risks. Participants also highlighted real‐time triage tools that link environmental data with emergency services as promising interventions to support at‐risk populations. Environmental monitoring systems can leverage Internet of Things (IoT)‐enabled climate‐health surveillance to integrate data on temperature, humidity, and pathogen levels. Other resource‐intensive solutions include predictive models that identify neighborhoods most vulnerable to heat or climate‐related health risks (e.g., Vieira Passos et al., 2024).

Participants proposed integrating existing applications related to air quality, disease risk, and extreme weather with satellite data and ML to deliver accessible information that could support protective behavioral change. The approach can be either creating a new web application or enhancing popular tools (e.g., Google Maps) to provide localized information at decision points. Key features of such tools should include translating synthesized information into clear health guidance, enabling user feedback, providing multilingual support, and fostering community partnerships. Ultimately, creating effective information tools needs partnerships across academia, government, industry, and user groups. A key research question is how information tools have been used in the past, whether and how they influence behavior, and which features most effectively motivate use.

Participants proposed government‐funded health monitoring to support national‐scale integration of environmental and health data into public health infrastructure, emphasizing the need for trained staff and effective communication systems to ensure these data inform timely action. In underserved areas, mobile cooling centers, ranging from buses with air‐conditioning to ice bag delivery and wellness checks, can bring services directly to those who need them most. Transportation assistance, such as subsidized rideshare programs during heatwaves, could bridge the critical gap between people and shelter. Key moments identified for user testing include deploying wellness checks during extreme weather events, launching mobile cooling resources, and piloting cooling applications that display nearby shelters in real time. These interventions should be evaluated for usability, uptake, and impact, especially among populations with limited mobility or digital access. Participants also noted that broader infrastructure dependencies, such as power availability during extreme weather events, can constrain the effectiveness of health‐focused interventions, though these issues were beyond the workshop's primary scope.

### 2.4 Reflection

To surface future research directions, the group began by reviewing select prototype ideas from design teams during the exhibition. Participants then engaged in reflective dialog, discussing what resonated and why, which aspects of the designs might be effective, and what technological innovations could be realistically envisioned. They reflected on what surprised them or expanded their thinking during the workshop and shared lessons learned and articulated potential research questions to guide deeper inquiry beyond the experience.

More broadly, promising future research directions included understanding how to encourage adoption of climate‐resilient behaviors (e.g., in response to poor air quality), leveraging advances in data science and Earth observation (e.g., satellites) for improved prediction, and identifying effective solutions to reduce impacts during extreme events. Building and deploying these tools will require coordinated partnerships across disciplines and sectors, including researchers, government and public health agencies, healthcare providers, education systems, industry, and community‐based organizations. Future research should also explore how information tools influence individual and community behavior and identify the design elements and collaborative strategies that most effectively support meaningful engagement and long‐term use.

### 2.5 Limitations and Lessons Learned

While the workshop demonstrated the potential of human‐centered design to reframe health research for threats driven by global change, several limitations should be acknowledged. Although participants were asked to adopt the perspectives of community members, policymakers, and practitioners, they remained researchers interpreting others' experiences from their own positions, introducing inherent biases shaped by their backgrounds, assumptions, and disciplinary lenses. Participation from nearly 100 researchers was strong, but the group was not fully representative across disciplines, sectors, or demographic backgrounds (e.g., overrepresentation from academia and limited participation from community‐based organizations or policy actors), which likely constrained the range of perspectives represented. Additionally, the compressed one‐day format constrained deeper engagement with user and affected‐actor personas and limited opportunities for iterative prototyping; a multi‐day format could support richer empathy‐building and refinement. Finally, without real‐time engagement with actual community stakeholders, agency and industry partners, decision‐makers, and affected public representatives, some key assumptions likely went unchallenged. Future workshops will integrate broader cross‐sector participation and a multi‐day format, including voices from frontline communities, policy and implementation sectors, and industry partners to strengthen collaboration and support the development of more actionable, inclusive, and equitable prototypes grounded in lived experiences.

## 3 Conclusion and Future Directions

**Figure 2:**
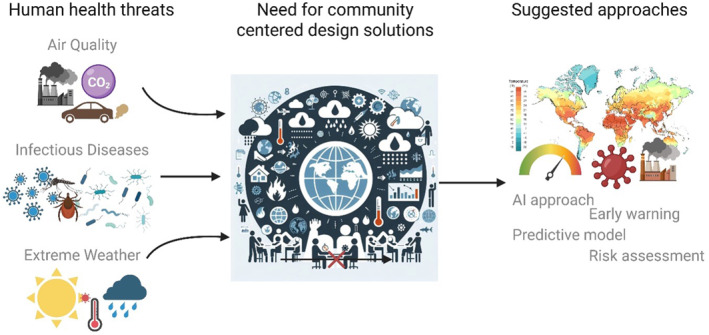
Conceptual framework linking human health threats (air quality, infectious diseases, and extreme weather) to the need for community‐centered design solutions, and to suggested approaches such as ML‐based methods, early warning, predictive modeling, and risk assessment. The center part of the figure was generated through Co‐pilot assistance.

The goal of this *One Earth One Health* workshop was to propose promising solutions and to train participants in human‐centered design, which has become a widely used approach in business innovation settings. This workshop provided an opportunity to introduce geoscience and human health researchers, many of whom are early career, to this approach. Rather than beginning with a hypothesis, participants focused on empathizing with selected end user and community perspectives, such as parents, first responders, and other affected groups. Participants role‐played these perspectives to better understand user priorities, constraints, concerns, and motivations related to global change, and to develop user stories. Sharing these stories helped participants broaden their understanding across perspectives. Participants then collaborated in breakout groups to propose prototype concepts, testing assumptions and identifying research questions relevant to potential implementation. Participants initially found it challenging to begin with a narrative rather than a scientific tool but ultimately embraced the approach. This process is summarized in Figure 2. Future efforts should incorporate post‐workshop evaluation and direct engagement with community members and other societal stakeholders to assess how well perceived needs and proposed solutions align with lived experiences and real‐world constraints, thereby better aligning research with societal needs.

Solution‐driven research requires more effort and less control over the finished product because researchers must co‐create and co‐design solutions with relevant non‐scientist actors, rather than relying solely on previous literature. The workshop participants were mostly early‐career researchers whose careers depend on demonstrating scholarship through peer‐reviewed publication. In general, scientists working at the intersection of geoscience and public health are not routinely trained in participatory co‐creation research methods, which are essential for communicating solutions to communities and supporting implementation. For this reason, expanded opportunities for inter‐ and transdisciplinary collaboration, education, and research training are needed. Specifically, cross‐disciplinary collaborations between researchers and societal actors outside of academia are needed to fill knowledge gaps and ensure that research outcomes are relevant, useful, and implementable in real‐world contexts.

A specific example where human‐centered solutions are useful is climate change policy. Most researchers aim to use research findings to inform the design, implementation, and evaluation of policy and community‐based solutions that reduce risks from climate‐related hazards and promote healthier environments. Unfortunately, gaps in understanding and trust persist among scientists, communities, policymakers, and other societal actors. For example, 60% of Americans understand that global warming is mostly human‐caused, and six‐in‐ten (60%) say stricter environmental laws and regulations are worth the cost (Leiserowitz et al., 2025; Pew Research Center, 2025). Even as nearly half of Americans report personally experiencing the effects of global warming, only 60% believe it is mostly human‐caused, indicating persistent gaps between lived experience and acceptance of scientific consensus (Leiserowitz et al., 2025). Global climate change was the lowest‐ranked threat among U.S. respondents in a Pew Research Center survey, while it is ranked highest by respondents from Belgium, France, the United Kingdom, and other high‐income countries (Poushter et al., 2022). Given this reality, progress in addressing the causes and consequences of global climate change in the U.S. has been slow, lagging other advanced nations. It is more important than ever for researchers in the U.S. to reflect and ask: What can be done differently? Should researchers critically examine how they conduct and communicate research findings, and consider where communication has gone wrong? It is worth noting that all prototypes proposed by participants after learning human‐centered approaches assume baseline public acceptance of climate change. Thus, researchers may best serve the public by pairing practical, co‐designed solutions with clear communication of the strong scientific consensus on anthropogenic climate change, while supporting informed decision‐making about how to respond.

The inaugural *One Earth*, *One Health* workshop provided an initial opportunity to apply human‐centered, process‐driven approaches in a workshop setting. While participants role‐played rather than working directly with communities, the workshop demonstrated how these methods can lay the groundwork for future collaborative solutions by helping researchers surface user needs, identify design assumptions, and translate interdisciplinary discussion into testable prototypes and research questions adaptable to other contexts.

## Conflict of Interest

The authors declare no conflicts of interest relevant to this study.

## Data Availability

Data were not used, nor created for this research.

## References

[gh270136-bib-0001] Agnello, D. M. , Anand‐Kumar, V. , An, Q. , de Boer, J. , Delfmann, L. R. , Longworth, G. R. , et al. (2025). Co‐creation methods for public health research—Characteristics, benefits, and challenges: A Health CASCADE scoping review. BMC Medical Research Methodology, 25(1), 60. 10.1186/s12874-025-02514-4 40050729 PMC11884017

[gh270136-bib-0002] Alotaibi, E. , & Nassif, N. (2024). Artificial intelligence in environmental monitoring: In‐depth analysis. Discover Artificial Intelligence, 4(1), 84. 10.1007/s44163-024-00198-1

[gh270136-bib-0003] Baseman, J. G. , Revere, D. , Painter, I. , Toyoji, M. , Thiede, H. , & Duchin, J. (2013). Public health communications and alert fatigue. BMC Health Services Research, 13(1), 295. 10.1186/1472-6963-13-295 23915324 PMC3751004

[gh270136-bib-0004] Bolan, S. , Padhye, L. P. , Jasemizad, T. , Govarthanan, M. , Karmegam, N. , Wijesekara, H. , et al. (2024). Impacts of climate change on the fate of contaminants through extreme weather events. Science of The Total Environment, 909, 168388. 10.1016/j.scitotenv.2023.168388 37956854

[gh270136-bib-0005] Chakraborty, T. , Qian, Y. , Li, J. , Leung, L. R. , & Sarangi, C. (2025). Daytime urban heat stress in North America reduced by irrigation. Nature Geoscience, 18(1), 57–64. 10.1038/s41561-024-01613-z

[gh270136-bib-0006] Colwell, R. R. (1996). Global climate and infectious disease: The cholera paradigm. Science, 274(5295), 2025–2031. 10.1126/science.274.5295.2025 8953025

[gh270136-bib-0007] Danasekaran, R. (2024). One health: A holistic approach to tackling global health issues. Indian Journal of Community Medicine, 49(2), 260–263. 10.4103/ijcm.ijcm_521_23 38665439 PMC11042131

[gh270136-bib-0008] Eilam, T. , Bose, P. , Carloni, L. P. , Cidon, A. , Franke, H. , Kim, M. A. , et al. (2024). Reducing datacenter compute carbon footprint by harnessing the power of specialization: Principles, metrics, challenges and opportunities. IEEE Transactions on Semiconductor Manufacturing, 37(4), 481–488. 10.1109/TSM.2024.3434331

[gh270136-bib-0009] Farooq, Z. , Rocklöv, J. , Wallin, J. , Abiri, N. , Sewe, M. O. , Sjödin, H. , & Semenza, J. C. (2022). Artificial intelligence to predict West Nile virus outbreaks with eco‐climatic drivers. The Lancet Regional Health ‐ Europe, 17, 100370. 10.1016/j.lanepe.2022.100370 35373173 PMC8971633

[gh270136-bib-0010] Filonchyk, M. , Peterson, M. P. , & Sun, D. (2022). Deterioration of air quality associated with the 2020 U.S. wildfires. Science of the Total Environment, 826, 154103. 10.1016/j.scitotenv.2022.154103 35218845

[gh270136-bib-0011] Gonen, E. (2019). Tim Brown, change by design: How design thinking transforms organizations and inspires innovation (2009). Markets, Globalization & Development Review, 4(2). 10.23860/MGDR-2019-04-02-08

[gh270136-bib-0012] Henderson, P. , Hu, J. , Romoff, J. , Brunskill, E. , Jurafsky, D. , & Pineau, J. (2020). Towards the systematic reporting of the energy and carbon footprints of machine learning. Journal of Machine Learning Research, 21(248), 1–43.34305477

[gh270136-bib-0013] Karavas, Z. , Karayannis, V. , & Moustakas, K. (2021). Comparative study of air quality indices in the European Union towards adopting a common air quality index. Energy & Environment, 32(6), 959–980. 10.1177/0958305X20921846

[gh270136-bib-0014] Kearney, G. D. , Namulanda, G. , Qualters, J. R. , & Talbott, E. O. (2015). A decade of environmental public health tracking (2002–2012). Journal of Public Health Management and Practice, 21(Supplement 2), S23–S35. 10.1097/PHH.0000000000000181 25621442 PMC5667361

[gh270136-bib-0015] Knapp, J. , Zeratsky, J. , & Kowitz, B. (2016). Sprint: How to solve big problems and test new ideas in just five days. Simon & Schuster.

[gh270136-bib-0016] Leiserowitz, A. , Maibach, E. , Rosenthal, S. , & Kotcher, J. (2025). Climate change in the American mind: Beliefs & attitudes, spring 2025. Yale Program on Climate Change Communication and George Mason University Center for Climate Change Communication. Retrieved from https://climatecommunication.gmu.edu/all/climate‐change‐in‐the‐american‐mind‐beliefs‐attitudes‐spring‐2025/CenterforClimateChangeCommunication

[gh270136-bib-0031] Lorsch, J. (2014). Archived: Hypothesis overdrive? National Institute of General Medical Sciences.

[gh270136-bib-0017] Ming, D. K. , Sangkaew, S. , Chanh, H. Q. , Nhat, P. T. H. , Yacoub, S. , Georgiou, P. , & Holmes, A. H. (2020). Continuous physiological monitoring using wearable technology to inform individual management of infectious diseases, public health and outbreak responses. International Journal of Infectious Diseases, 96, 648–654. 10.1016/j.ijid.2020.05.086 32497806 PMC7263257

[gh270136-bib-0018] Mora, C. , McKenzie, T. , Gaw, I. M. , Dean, J. M. , von Hammerstein, H. , Knudson, T. A. , et al. (2022). Over half of known human pathogenic diseases can be aggravated by climate change. Nature Climate Change, 12(9), 869–875. 10.1038/s41558-022-01426-1 PMC9362357 35968032

[gh270136-bib-0019] Olawade, D. B. , Wada, O. Z. , Ige, A. O. , Egbewole, B. I. , Olojo, A. , & Oladapo, B. I. (2024). Artificial intelligence in environmental monitoring: Advancements, challenges, and future directions. Hygiene and Environmental Health Advances, 12, 100114. 10.1016/j.heha.2024.100114

[gh270136-bib-0020] Oviedo‐Trespalacios, O. , Peden, A. E. , Cole‐Hunter, T. , Costantini, A. , Haghani, M. , Rod, J. E. , et al. (2023). The risks of using ChatGPT to obtain common safety‐related information and advice. Safety Science, 167, 106244. 10.1016/j.ssci.2023.106244

[gh270136-bib-0021] Pew Research Center . (2025). Support for stricter environmental regulations outweighs opposition in a majority of states. Pew Research Center. Retrieved from https://www.pewresearch.org/short‐reads/2025/05/19/support‐for‐stricter‐environmental‐regulations‐outweighs‐opposition‐in‐a‐majority‐of‐states/#:~:text=Americans'%20views%20on%20federal%20environmental,to%20curb%20global%20climate%20change

[gh270136-bib-0022] Poushter, J. , Fagan, M. , & Gubbala, S. (2022). Climate change remains top global threat across 19‐country survey. Pew Research Center.

[gh270136-bib-0023] Romanello, M. , Di Napoli, C. , Drummond, P. , Green, C. , Kennard, H. , Lampard, P. , et al. (2022). The 2022 report of the Lancet Countdown on health and climate change: Health at the mercy of fossil fuels. The Lancet, 400(10363), 1619–1654. 10.1016/S0140-6736(22)01540-9 PMC7616806 36306815

[gh270136-bib-0024] Savoia, E. , Lin, L. , & Viswanath, K. (2013). Communications in public health emergency preparedness: A systematic review of the literature. Biosecurity and Bioterrorism: Biodefense Strategy, Practice, and Science, 11(3), 170–184. 10.1089/bsp.2013.0038 24041193 PMC3778998

[gh270136-bib-0025] Seshadri, D. R. , Davies, E. V. , Harlow, E. R. , Hsu, J. J. , Knighton, S. C. , Walker, T. A. , et al. (2020). Wearable sensors for COVID‐19: A call to action to harness our digital infrastructure for remote patient monitoring and virtual assessments. Frontiers in Digital Health, 2, 8. 10.3389/fdgth.2020.00008 34713021 PMC8521919

[gh270136-bib-0026] Tiller, R. G. , Destouni, G. , Golumbeanu, M. , Kalantari, Z. , Kastanidi, E. , Lazar, L. , et al. (2021). Understanding stakeholder synergies through system dynamics: Integrating multi‐sectoral stakeholder narratives into quantitative environmental models. Frontiers in Sustainability, 2, 701180. 10.3389/frsus.2021.701180

[gh270136-bib-0027] Topol, E. J. (2019). High‐performance medicine: The convergence of human and artificial intelligence. Nature Medicine, 25(1), 44–56. 10.1038/s41591-018-0300-7 30617339

[gh270136-bib-0028] U.S. Environmental Protection Agency . (2025). Community multiscale air quality (CMAQ) model. Retrieved from https://www.epa.gov/cmaq

[gh270136-bib-0029] Vieira Passos, M. , Kan, J.‐C. , Destouni, G. , Barquet, K. , & Kalantari, Z. (2024). Identifying regional hotspots of heatwaves, droughts, floods, and their co‐occurrences. Stochastic Environmental Research and Risk Assessment, 38(10), 3875–3893. 10.1007/s00477-024-02783-3

[gh270136-bib-0030] Wallerstein, N. , & Duran, B. (2010). Community‐based participatory research contributions to intervention research: The intersection of science and practice to improve health equity. American Journal of Public Health, 100(Suppl 1), S40–S46. 10.2105/AJPH.2009.184036 20147663 PMC2837458

